# Burnout syndrome and its prevalence in primary care nursing: a systematic review and meta-analysis

**DOI:** 10.1186/s12875-018-0748-z

**Published:** 2018-05-10

**Authors:** Carolina S. Monsalve-Reyes, Concepción San Luis-Costas, Jose L. Gómez-Urquiza, Luis Albendín-García, Raimundo Aguayo, Guillermo A. Cañadas-De la Fuente

**Affiliations:** 1 Departamento de Ciencias Sociales, Universidad Católica de La Santísima Concepción, Concepción, Chile; 2 Doctorado en Psicología de la Salud, Facultad de Psicología, Universidad Nacional de Educación a Distancia (UNED), Madrid, Spain; 3 Spanish University of Distance Education, Madrid, Spain; 4 Nursing Department, University of Granada, Granada, Spain; 5 Andalusian Health Service and University of Granada, Granada, Spain; 6 Psychology Department, University of Granada, Granada, Spain; 7 Nursing Department, University of Granada, Granada, Spain; 8 Faculty of Health Sciences, University of Granada, Cortadura del Valle Street S/N, 51001 Ceuta, Spain

**Keywords:** Burnout, Primary care nursing, Nursing, Family nursing, Meta-analysis, Epidemiology, Prevalence

## Abstract

**Background:**

burnout syndrome is a significant problem in nursing professionals. Although, the unit where nurses work may influence burnout development. Nurses that work in primary care units may be at higher risk of burnout. The aim of the study was to estimate the prevalence of emotional exhaustion, depersonalization and low personal accomplishment in primary care nurses.

**Methods:**

We performed a meta-analysis. We searched Pubmed, CINAHL, Scopus, Scielo, Proquest, CUIDEN and LILACS databases up to September 2017 to identify cross-sectional studies assessing primary care nurses’ burnout with the Maslach Burnout Inventory were included. The search was done in September 2017.

**Results:**

After the search process, *n* = 8 studies were included in the meta-analysis, representing a total sample of *n* = 1110 primary care nurses. High emotional exhaustion prevalence was 28% (95% Confidence Interval = 22–34%), high depersonalization was 15% (95% Confidence Interval = 9–23%) and 31% (95% Confidence Interval = 6–66%) for low personal accomplishment.

**Conclusions:**

Problems such as emotional exhaustion and low personal accomplishment are very common among primary care nurses, while depersonalization is less prevalent. Primary care nurses are a burnout risk group.

**Electronic supplementary material:**

The online version of this article (10.1186/s12875-018-0748-z) contains supplementary material, which is available to authorized users.

## Background

The development of burnout among healthcare professionals has been widely studied in recent years, since the large number of stress-inducing factors in the hospital environment heightens the risk of presenting burnout syndrome [1–3]. In addition, relationships and continued contact with patients and their families can be difficult, which fosters the development of chronic stress that can provoke burnout among healthcare staff [4].

The burnout syndrome has been extensively studied, even though the most widely accepted definition of burnout is that proposed by Maslach & Jackson [5], who identified it as a three-dimensional syndrome involving emotional exhaustion (EE), cynical treatment and negative thoughts towards patients and the healthcare team (or depersonalisation, DP), and a low degree of personal accomplishment (PA) regarding the own work performed. The study of burnout is important because its negative effects can impact both on the professional who suffers it, causing different signs and symptoms [6], and also on the health institution itself, by increasing staff absenteeism, and on the quality of care provided by increasing medical errors and diminishing patient safety [7, 8].

In hospital settings, nurses are among the professionals most affected by burnout [9] and for this reason numerous studies have been conducted to identify protective factors and elements of risk. For example, some sociodemographic factors such as age, gender or marital status and its influence have been assessed [10, 11]. Psychological factors like the big five personality traits [12] or occupational factors, such as job seniority or job satisfaction, have been also studied [13–15].

However, one key factor that may be associated with burnout syndrome is the hospital service in which nurses work; the tasks performed and the role played by the healthcare staff, as well as the type of patients treated, all vary according to the type of service provided, and this difference could influence the development of the syndrome. For example, nurses working in oncology [16], accident and emergency units [17] or intensive care [18], due to their different daily tasks, are likely to experience different levels and prevalence rates of burnout.

Primary healthcare units differ in many respects from the attention provided in hospital units, in that preventive and remedial treatment is provided for chronic diseases, to pre-assigned groups of patients. Primary healthcare is provided in the community itself, and may take place over a prolonged period [19]. By contrast, in the hospital environment the medical treatment is of a shorter-term nature, and there is greater variability among the patients. Although burnout and its risk factors in nursing primary care professionals, such as age, job seniority, anxiety and depression, have been studied previously [20], the prevalence results reported by the studies vary widely, with some authors reporting a high EE of 5,2% [21] while others report 31,3% [22]. Similar situations occur with high DP and low PA, where some authors find 92,8% of the sample with low PA [23] and others find a 4,3% of the sample with low PA [22]. So, it is difficult to ascertain the real impact of burnout syndrome on primary healthcare nurses. To our knowledge, no previous meta-analysis has been undertaken to address this question, as has been done in the case of nurses working in services such as Accident & Emergency [24] or oncology [25].

Taking into account the above considerations, we aimed to conduct a systematic review and meta-analysis of the prevalence of high levels of EE, high levels of DP and low levels of PA among primary care nurses. Thus, the question that guided this meta-analysis was: What is the prevalence rate of high EE, high DP and low PA in primary care nurses?

## Method

This study consists of a meta-analysis, performed in accordance with the PRISMA recommendations [26].

### Literature search and study selection

The following search terms were used: “burnout AND primary care nursing”, “burnout AND family nursing”, “burnout AND community health nursing” and “burnout AND district nursing”. The search was carried out in September 2017, consulting the following research databases: Pubmed, CUIDEN, CINAHL, LILACS, Proquest, Scopus and Scielo.

Conditions for inclusion were that the papers should be primary studies, of a quantitative type, based on a sample of primary healthcare nurses and providing data on the prevalence of any burnout dimension (EE, DP or PA), measured by the Maslach Burnout Inventory (MBI) [5]. The studies should have been published in English, Spanish or Portuguese, but no restriction was placed regarding the date of publication.

The search and study selection process was conducted by two members of the research team, working independently, to ensure the reliability of the process. If they disagreed regarding the inclusion or otherwise of a paper, a third member of the research team was consulted. The selection process involved an initial reading, of the title and abstract. The papers initially selected were then read in full, and those considered a priori suitable for inclusion were then subjected to a critical reading to detect possible methodological bias. From the papers finally selected, backward and forward citation checking was then performed.

### Critical reading

All of the studies included were cross-sectional, and their methodological quality was evaluated by the checklist suggested by Ciapponi [27], using the items corresponding to the studies internal validity: numbers 2, 3, 4, 5, 6, 11, 12, 13, 14,2 15, 16, 17 and 18. The critical reading results are shown in Additional file 1.

### Data coding

The following study variables were collected: a) surname of the first author; b) date of publication; c) language of the study; d) country where the research was carried out; e) study methodology; f) type of sampling; g) MBI type (Human Services Survey vs. General Survey); h) sample of primary healthcare nurses; i) sample of primary healthcare nurses with high EE; j) sample of primary care nurses with high DP; k) sample of primary healthcare nurses with low PA. These data were compiled using a Coding Manual.

The intraclass correlation coefficient and Cohen’s kappa coefficient were calculated to evaluate the reliability of the data coding, producing mean values of 0.99 (minimum = 0.98, maximum = 1) and 0.97 (minimum = 0.95, maximum = 1), respectively.

### Data analysis

Data analysis was performed using StatsDirect software, with the meta-analysis package, and the possibility of publication bias was tested by Egger’s linear regression method. Finally, a sensitivity analysis was carried out to detect whether exclusion of any of the studies would have produced significant changes in the results obtained.

The prevalence and the confidence interval of each dimension of burnout were calculated by three independent random-effects meta-analyses. Sample heterogeneity was analysed with the Cochran Q test and the *I*^*2*^ index.

## Results

**Fig. 1:**
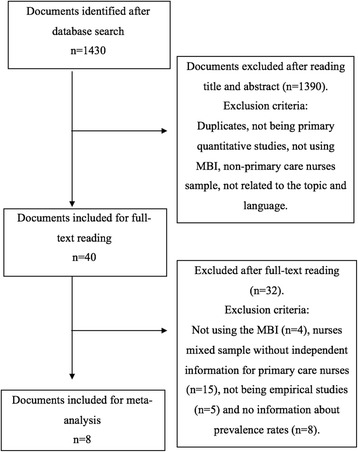
Documents search flow diagram

The search process initially obtained *n* = 1430 results, which after reading title and abstract, full-text and the application of the inclusion and exclusion criteria were reduced to *n* = 8 for high EE and *n* = 7 for high DP and low PA [21–23, 28–32]. The selection process is illustrated in Fig. 1.

All of the included studies were transversal and descriptive, and used the HSS version of the MBI. Only one study used random sampling, and 75% were conducted in Europe. The characteristics of these studies are listed in Table 1.

**Table 1:** Characteristics of included studies (*n* = 8)

Author, year. Country	Study type; Sampling method	Type of MBI	Sample size (Females%; Response rate%)	n with high EE	n with high DP	n with low PA
Das Merces et al., 2016. Brasil [28]	O; Intentional	HSS	*n* = 28 (100%; 90,32%)	8	6	13
Faura et al., 1995. Spain [29]	O; Intentional	HSS	*n* = 116 (90.5%; ND)	27	–	–
Hayter, 1999. England [30]	O; Intentional	HSS	*n* = 30 (80%; 94%)	8	0	6
Holmes et al., 2014. Brasil [31]	O; Random	HSS	*n* = 45 (100%; 100%)	24	5	5
Navarro-Gonzalez et al., 2005. Spain [21]	O; Intentional	HSS	*n* = 178 (73,3%; 48%)	28	33	84
Soto Cámara et al., 2005. Spain [23]	O; Intentional	HSS	*n* = 208 (86.5%: 83,87%)	60	67	193
Tomas-Sabado et al., 2010. Spain [32]	O; Intentional	HSS	*n* = 160 (89.7%; 67%)	35	19	14
Vila Faguera et al., 2015. Spain [22]	O; Intentional	HSS	*n* = 345 (80.2%; 44.5%)	108	51	15

*DP* Depersonalization, *EE* Emotional exhaustion, *HSS* Human Services Survey, *MBI* Maslach Burnout Inventory, *ND* No Data, *O* Observational, *PA* Personal Accomplishment

The sensitivity analysis performed, of the influence of each study on the overall result, revealed no statistically significant changes in the prevalence rates. Neither was any significant publication bias detected, being Egger’s linear regression score 1.66 (*p* = 0.43) for high EE, 1.09 (*p* = 0.77) for high DP and 5.29 (*p* = 0.67) for low PA.

Cochran’s Q value was 33.10 (*p* < 0.001) for EE, 46.31 (p < 0.001) for DP and 697.45 (p < 0.001) for low PA. The *I*^*2*^ index revealed a high level of heterogeneity, at 78.9% for EE, 87% for DP and 99.1% for low PA.

**Fig. 2:**
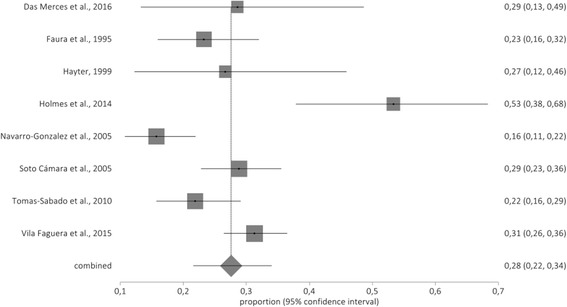
Forestplot of high emotional exhaustion

The total population for the meta-analysis was composed of *n* = 1110 primary care nurses. The prevalence rate obtained for high emotional exhaustion was 28% (95% CI: 22–34%). Figure 2 shows the forestplot of high EE.

**Fig. 3:**
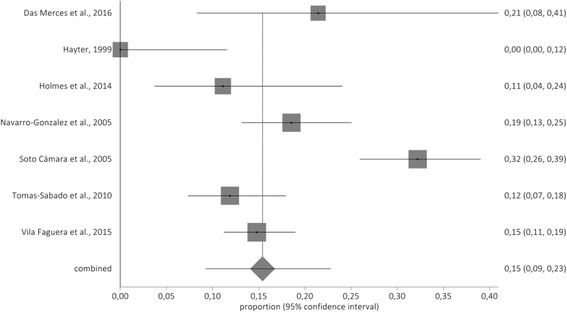
Forestplot of high depersonalization

In depersonalization dimension the prevalence rate was 15% (95% CI: 9%–23%). The forestplot of high DP is shown in Fig. 3.

**Fig. 4:**
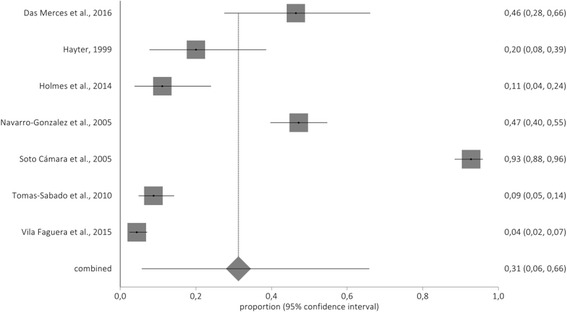
Forestplot of low personal accomplishment

Finally, a 31% (95% CI: 6–66%) was found for low personal accomplishment. Figure 4 show illustrate the forestplot of low PA.

## Discussion

Our meta-analysis shows that low personal accomplishment is the most widely affected dimension of burnout in primary care nurses, being present in 31% of the sample. This is followed by emotional exhaustion, which was observed in 28% of the nurses in the sample. The lowest level of prevalence corresponded to depersonalisation, which affected 15% of these nurses.

To our knowledge, no previous meta-analysis has been performed regarding the prevalence of burnout in primary care nurses. The number of studies included in the present meta-analysis is lower than in those with A&E nurses [24] or oncology nurses [25]. Meta-analytical studies of the prevalence of burnout among A&E nurses have reported values similar to our own for EE and low PA, with prevalence rates of 31% and 29%, respectively. However, these previous papers measured a much higher presence of DP (36%) than in our sample of primary care nurses [24]. On the other hand, a meta-analysis focusing on burnout among oncology nurses observed higher levels of EE, lower levels of DP and PA, than in the primary care nurses in our sample [25]. Nevertheless, it is foreseeable that primary care nurses will experience less DP than A&E nurses, because they often follow the evolution of chronic patients for years, perhaps visiting patients in their homes and in their community. This situation favours empathy and the formation of a close relationship with the patient, which is of crucial importance to the quality of care [33].

The important degree of EE and low PA observed among primary care nurses may be due, among other factors, to their sometimes encountering difficult patients, who can be very demanding and may continually seek medical attention, necessary or otherwise. Such persons disrupt the routines of the service and have a negative impact on medical professionals [34–36]. In addition, the importance that primary care is gaining in health services, and the growing demand for services in this area, forces nurses to meet the new challenges and requests that are generated, adding more pressure to their work [37]. To all this, we must add the difficulties of nursing work, such as contact with vulnerable people and families in consultation and in patients´ homes [38], the increasing workload, or the lack of control over their own work environment [39], which may favour EE and decrease PA.

Some limitations of this study should be acknowledged. The number of studies included is low, because not all studies of burnout in primary care nurses provide prevalence data. Moreover, the studies included are all cross-sectional and descriptive, which accounts for their low level of evidence (although this design is usually considered appropriate for prevalence studies). Furthermore, the included studies, despite meeting almost all the items of the critical reading guide, have small sample sizes. To this, we must add the high heterogeneity found in the results, which may be due to the different countries where the studies have been done, because the healthcare systems of each country have different workplace conditions, salary, shifts and competencies for the nursing workforce [40], which may influence nurses´ burnout and should be taken into account when interpreting the results.

Future research in this area should include a longitudinal evaluation of the sociodemographic, occupational, and psychological factors that may influence the development of burnout syndrome among primary care nurses. It would be interesting to examine interventions aimed at preventing and/or reducing emotional exhaustion in this occupational group. Finally, in view of the results obtained in this meta-analysis, action should be taken to enhance feelings of personal accomplishment among primary care nurses, for example by fostering professional empowerment, by establishing primary care nurses’ groups so they can express their feeling [41], promoting leadership capabilities and positive psychological capacities [42] or improving the workplace conditions.

## Conclusions

Problems such as emotional exhaustion and low personal accomplishment are very common among primary care nurses from the countries included in the meta-analysis, with a prevalence between 22 and 34% and around 31% respectively. Depersonalisation is less prevalent in this population, at around 15%, which indicates that primary care nurses included in the meta-analysis relate well with their patients.

## Additional file

Additional file 1: Critical reading. Description of data: Studies critical reading results (DOCX 18 kb)

## Abbreviations

CI: Confidence Interval
DP: Depersonalization
EE: Emotional Exhaustion
MBI: Maslach Burnout Inventory
PA: Personal Acomplishment
