# Supplementary material for: Burnout syndrome and its prevalence in primary care nursing: a systematic review and meta-analysis
Source: BMC Fam Pract. 2018 May 10;19:59. doi: 10.1186/s12875-018-0748-z (PMC5944132; doi:10.1186/s12875-018-0748-z)
Supplement: Supplementary file 1 — Critical reading. Description of data: Studies critical reading results (DOCX 18 kb) [file 12875_2018_748_MOESM1_ESM.docx]

**Additional file 1. Critical reading [27]**

| Item | 1 | 2 | 3 | 4 | 5 | 6 | 7 | 8 |
| --- | --- | --- | --- | --- | --- | --- | --- | --- |
| Inclusion and exclusion criteria and selection methods are indicated. | Y | Y | Y | Y | Y | Y | Y | Y |
| Selection criteria are adequate to answer the study aim. | Y | Y | Y | Y | Y | Y | Y | Y |
| The study population, define by the selection criteria, contains an adequate spectrum of the population of interest. | Y | Y | Y | Y | Y | Y | Y | Y |
| A sample size estimation, confidence level or statistical of the sample was done. | Y | N | N | N | N | N | N | N |
| The number of potentially eligible persons is reported, those initially selected, those who accept and those who finally participate. | Y | Y | Y | Y | Y | Y | N | Y |
| The variables of exposure/intervention, result, confusing or modifying are clearly stated | Y | Y | Y | Y | Y | Y | Y | Y |
| The main variables have an adequate conceptual and operational definition | Y | Y | Y | Y | Y | Y | Y | Y |
| The instruments for measuring the main variables have known and adequate validity and reliability | Y | Y | Y | Y | Y | Y | Y | Y |
| The measurement and collection techniques of the main variables are sufficiently described | Y | Y | Y | Y | Y | Y | Y | Y |
| The statistical analysis was determined from the beginning of the study. | Y | Y | Y | Y | Y | Y | Y | Y |
| The statistical tests used are specified and are adequate | Y | Y | Y | Y | Y | Y | Y | Y |
| Participants and data lost were correctly analysed. | Y | Y | Y | Y | Y | Y | Y | Y |
| The main elements of possible confusion in design and analysis were taken into account | Y | Y | Y | Y | Y | Y | Y | Y |
| Methodological Quality | H | H | H | H | H | H | M | H |

*Note*: H= High; L= Low; M= Medium; N= No; Y= Yes; 1= Reference number 21; 2= Reference number 22; 3= Reference number 23; 4= Reference number 28; 5= Reference number 29; 6= Reference number 30; 7= Reference number 31; 8= Reference number 32.
